# A Case of Fulminant Fat Embolism Syndrome With Very Early Onset After Femoral Neck and Sacral Fractures

**DOI:** 10.7759/cureus.35911

**Published:** 2023-03-08

**Authors:** Seigo Kimura, Ryokichi Yagi, Fumihisa Kishi, Daiji Ogawa, Keiichi Yamada, Hirokatsu Taniguchi, Masahiko Wanibuchi

**Affiliations:** 1 Department of Neurosurgery, Kouzenkai Yagi Neurosurgical Hospital, Osaka, JPN; 2 Department of Neurosurgery, Osaka Medical and Pharmaceutical University, Osaka, JPN

**Keywords:** early onset, fulminant fat embolism syndrome, consciousness, pneumonia, fracture

## Abstract

Fulminant fat embolism syndrome (FES) occurring within 1 h after trauma is extremely rare. We report a case of fulminant FES that developed hyperacute nature after a traumatic injury. A 66-year-old woman was injured when she fell approximately 1.5 m down the stairs. She was rushed to our hospital. One minute after arrival, which was 49 min after the injury, her consciousness and respiratory status deteriorated. Thoracoabdominal and pelvic computed tomography revealed preexisting interstitial pneumonia, a left femoral neck fracture, and a left sacral fracture. Head magnetic resonance imaging (diffusion-weighted imaging) showed diffuse high-signal areas and susceptibility-weighted imaging showed diffuse small perivascular of perivascular hemorrhages. She was diagnosed with fulminant FES. After conservative treatment, she was transferred to a rehabilitation hospital with a Glasgow Coma Scale (GCS) of 8 and a modified Rankin Scale of 5 on Day 45. The possibility of fulminant FES should be considered a cause of early impaired consciousness after a fracture.

## Introduction

Fat embolism syndrome (FES) is a syndrome in which fat emboli occur in the lungs, brain, or skin after a fracture of the femur or other long bones [1]. FES develops within an average of 48.5 hours after a long bone fracture [2]. Among FES, those that develop rapidly after an injury are known as fulminant FES and are considered to have a severe course [3]. Fulminant FES occurring within 1 h of trauma is extremely rare. Herein, we report a case of fulminant FES that developed very early after a femoral neck fracture and sacral fracture.

## Case presentation

**Figure 1:**
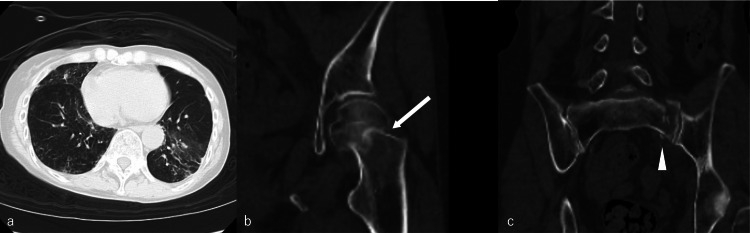
Thoracoabdominal and pelvic computed tomography at the time of the traumatic injury a) Preexisting interstitial pneumonia, b) Left femoral neck fracture (white arrow), c) Left sacral fracture (white arrowhead)

A 66-year-old woman was injured when she fell approximately 1.5 m down the stairs at her home. She was unable to walk due to pain in her left lower limb, and her family called for emergency medical assistance immediately after the injury. She was rushed to our hospital and was initially treated by an orthopedic surgeon. Her medical history included chronic rheumatoid arthritis and interstitial pneumonia. When she came to our hospital, her consciousness was clear and no obvious neurological abnormalities were noted. Her temperature was 36.6℃, her pulse was 94/min, her blood pressure was 143/74 mmHg, and her oxygen saturation (SpO2) was 95% (room air). One minute after arrival, 49 min after onset, the patient was referred to neurosurgery due to a decreased level of consciousness. Her Glasgow Coma Scale (GCS) was 6 (E1, V1, M4), she had no pupillary irregularity, and she exhibited flaccid paralysis in all extremities. Her vital signs were as follows: pulse 85/min, blood pressure 95/45 mmHg, and SpO2 93%. O2 3 l was started. Electrocardiogram showed a normal sinus rhythm. A blood test revealed the following: RBC 3.81×106/μl, Hb 12.1g/dl, hematocrit (Ht) 37.2%, WBC 13900/μl (Neu 70.8%, Lym 26.9%, Mo 2.2%, Eo 0.1%, Ba 0%), platelet 265×103/μl, prothrombin time-international normalized ratio (PT-INR) 1.01, activated partial thromboplastin time (aPTT) 26.3 s, total protein (TP) 6.9 g/dl, Alb 4.0 g/dl, aspartate aminotransferase (AST) 25 U/L, alanine transaminase (ALT) 16 U/L, creatine kinase (CK) 82 U/L, lactate dehydrogenase (LDH) 410 U/L, cholinesterase (ChE) 317 U/L, creatinine (Cr) 0.56 mg/dl, blood urea nitrogen (BUN) 11.5 mg/dl, glucose 161 mg/dl, Na 140 mEq/L, K 3.2 mEq/L, Cl 103 mEq/L, and CRP 4.5 mg/dl. A blood gas examination revealed the following: pH 7.42, partial pressure of carbon dioxide (PaCO2) 30.5 mmHg, partial pressure of oxygen (PaO2) 71.0 mmHg, HCO3- 21.7 mmol/L, SpO2 94.6%, and base excess (BE) −3.7 mmol/L at 3 l O2 administration (Table 1). Thoracoabdominal and pelvic computed tomography (CT) showed preexisting interstitial pneumonia, a left femoral neck fracture, and a left sacral fracture (Figures 1a-1c).

**Table 1:** A blood test on admission prothrombin time-international normalized ratio (PT-INR); activated partial thromboplastin time (aPTT); total protein (TP); aspartate aminotransferase (AST); alanine transaminase (ALT); creatine kinase (CK); lactate dehydrogenase (LDH); cholinesterase (ChE); creatinine (Cr); blood urea nitrogen (BUN); partial pressure of carbon dioxide (PaCO2); partial pressure of oxygen (PaO2); oxygen saturation (SpO2); base excess (BE)

Complete blood count	Test result	Normal range	Biochemical examination	Test result	Normal range	Air blood gas (O2 3l)	Test result	Normal range
WBC (10^3^/μl)	13.9	3.3 - 8.4	TP (g/dl)	6.9	6.5 - 8.2	pH	7.42	7.38 - 7.42
Neu (%)	70.8	37 - 74	Alb (g/dl)	4.0	3.7 - 5.5	PaCO2 (mmHg)	30.5	38 - 45
Lym (%)	26.9	18 - 50	AST (U/l)	25	10 - 40	PaO2 (mmHg)	71.0	75 - 107
Mo (%)	2.2	2-8	ALT (U/l)	16	5 - 45	HCO3- (mmol/l)	21.7	22 - 26
Eo (%)	0.1	0 - 6	CK (U/l)	82	50 - 210	SpO2 (%)	94.6	95 - 100
Ba (%)	0	0 - 2	LDH (U/l)	410	120 - 245	BE (mmol/l)	-3.7	-2 - 2
RBC (×10^6^/μl)	3.81	3.8 - 5.0	ChE (U/l)	317	198 - 452			
Hb (g/dl)	12.1	11.3 - 15.2	Cre (mg/dl)	0.56	0.46 - 0.79	COVID		
Plt (×10^4^/μl)	26.5	12.0 - 34.0	BUN (mg/dl)	11.5	8.0 - 20.0	Antigen	-	-
			Glu (mg/dl)	161	7 - 109	PCR	-	-
Blood Coagulation Test			Na (mEq/l)	140	135 - 147			
PT-INR	1.01	0.9 - 1.1	K (mEq/l)	3.2	3.5 - 5.0			
aPTT(sec)	26.3	25 - 40	Cl (mEq/l)	103	98 - 108			
			CRP (mg/dl)	4.5	0 - 0.3			

**Figure 2:**
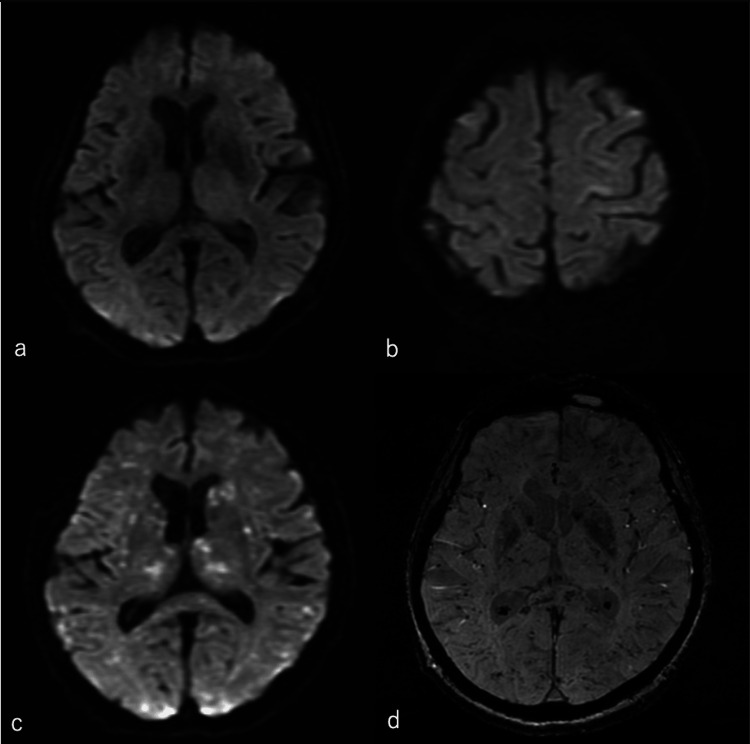
Day 0 and Day 2 head magnetic resonance imaging a,b) Diffusion-weighted imaging on Day 0: slight punctate hyperintensities. c) Diffusion-weighted imaging on Day 2: scattered high signal. d) Susceptibility-weighted imaging on Day 2: scattered low signal.

Head magnetic resonance imaging diffusion-weighted imaging (MRI DWI) showed slight punctate hyperintensities (Figures 2a-2b). Based on the patient's impaired consciousness after the fracture, she was diagnosed with fulminant FES and conservative treatment was administered. After admission to the intensive care unit, her temperature rose to 38.9℃, and she was tachycardic with a pulse rate of 128/min. SpO2 dropped to the 80% range, so 10 l O2 was administered. Drug therapy with sulbactam sodium/ampicillin sodium (SBT/ABPC) 3 g/day, levetiracetam 1000 mg/day prophylactically for seizure, and edaravone 60 mg/day was started. A head MRI DWI performed 2 h after the loss of consciousness revealed a slightly more diffuse high signal. On Day 2, a head MRI revealed clearly scattered high signals on DWI and multiple low signals on susceptibility-weighted imaging (Figures 2c-2d).

**Figure 3:**
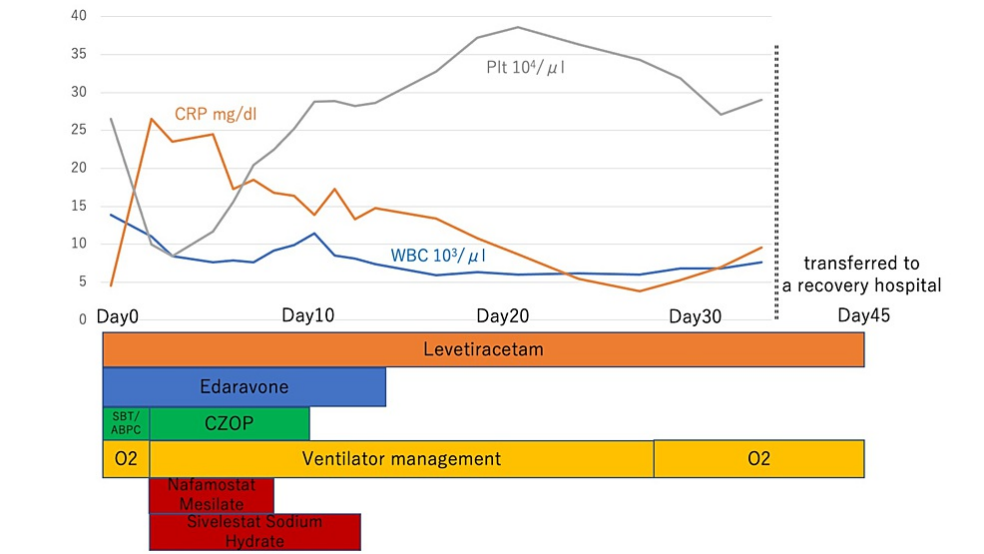
Course of treatment After admission, O2 10l was administered and sulbactam sodium/ampicillin sodium (SBT/ABPC), levetiracetam, and edaravone were started. On Day 2, respiratory status deteriorated and platelets decreased. Ventilatory management was started, antibiotics were changed to cefozopran (CZOP), and nafamostat mesylate and sivelestat sodium hydrate were started. Her general condition gradually improved, and she was transferred to a rehabilitation hospital with a Glasgow Coma Scale of 8 and a modified Rankin's score of 5 on Day 45.

SpO2 dropped to 80% despite administration of 10 l O2 on the same day, chest CT showed worsening pneumonia, and blood sampling showed a rapid drop in platelets. Ventilatory management was started, antibiotics were changed to cefozopran (CZOP) 2 g/day, and nafamostat mesylate (200 mg/day) and sivelestat sodium hydrate (50 mg/day) were started. After orthopedic consultation regarding the fracture, conservative treatment was first chosen due to unstable vitals and low platelet counts. On Day 10, slight hemorrhagic spots were observed in the anterior thoracic region. On Day 12, her GCS was 7 (E2, V1, M4), her vital signs stabilized, her platelet count improved, and a tracheostomy was performed. Because her general condition improved, we consulted an orthopedic surgeon regarding the indications for surgery for the left femoral neck fracture and left sacral fracture. Conservative treatment was chosen due to residual impaired consciousness, inability to expect improvement in activities of daily living after surgery, and the risk of FES recurrence due to the surgery. On Day 45, she was transferred to a rehabilitation hospital with a GCS of 8 (E3, V1, M4) and a modified Rankin Scale 5 (Figure 3).

## Discussion

FES is triggered by femoral or other long bone fractures or intramedullary nail surgery. Fat droplets enter the circulatory system, causing fat embolisms in the lungs, brain, and skin, resulting in respiratory symptoms, central nervous system symptoms, and cutaneous petechial hemorrhages [1]. FES occurs in 1%-22% of all long bone fractures, and no effective treatment has been established. The mortality rate for FES ranges from 7% to 20% [4]. Our patient had three of the major Gurd and Wilson criteria for the diagnosis of FES [5]: hemorrhagic plaques, respiratory failure, and impaired consciousness unrelated to head trauma. In addition, tachycardia, fever, decreased hemoglobin, and thrombocytopenia were noted, fulfilling many of the diagnostic criteria for FES. Sevitt et al. classified FES into three types [3]. The first type is fulminant FES, which progresses rapidly and can be fatal. This type is clinically difficult to detect because petechial hemorrhage is often unrecognized due to rapid death. The second type is classic FES, characterized by the triad of dyspnea, neurologic symptoms, and petechial hemorrhage [6,7]. Classic FES usually presents within 12-72 h after trauma. The third type is incomplete or partial FES, which may or may not present with typical symptoms and has a low mortality rate [3]. Survival time correlates with the severity of FES [8]. Our case was classified as fulminant FES with a high mortality rate because it occurred only 49 min after injury.

The two theories for the pathogenesis of FES include mechanical embolization of free fat droplets and chemical injury caused by tissue damage from cytokines and free fatty acids [9]. Since the onset of this case was extremely short, we speculate that mechanical embolization caused by fat droplets was most likely. The characteristic imaging findings were a “starfield pattern” of diffuse high-signal areas on MRI (DWI) and a small punctate of perivascular hemorrhage on susceptibility-weighted imaging [10,11]. In our case, both of these findings were present.

Symptomatic treatment, including respiratory, circulatory, and intracranial pressure control, is the mainstay of treatment. Corticosteroids, urinastan, sivelestat sodium hydrate, and edaravone may be effective, but their efficacy in the strict sense has not been established [12]. Although early stabilization of the fracture site is an important preventive measure [5], in the present case, the onset of the FES occurred very early before orthopedic surgery was performed. The patient's respiratory condition was unstable and thrombocytopenic occurred early during the onset of the disease. Therefore, an invasive, hematological procedure was judged to be highly risky. The patient's consciousness remained impaired even after her general condition stabilized. Thus, surgery could not be expected to improve her activities of daily living, and surgery could have caused a recurrence of FES. Therefore, conservative treatment was chosen. The patient did not experience any FES recurrence.

Among the reported fulminant FES cases, seven cases [13-18], including our case, were identified that developed less than 3 h after injury, with our case being the earliest after injury (Table 2). Most of the fulminant FES cases were severely ill. Another possible mechanism for cerebral fat embolization is that pulmonary fat embolization increases right atrial pressure, causing the leftover patent foramen ovale (PFO) to open, resulting in a right-to-left shunt, which allows fat droplets to flow into the arterial side and cause cerebral infarction [19]. However, none of the seven patients with fulminant FES showed PFO patency by echocardiography or transesophageal echocardiography, and none of the reports described a specific mechanism for the early onset of the disease. Past reports suggested that fat droplets liberated into the blood due to the fracture may have dispersed into the lungs, and fat droplets that did not stay in the lungs may have passed through the pulmonary capillary layer or intrapulmonary physiologic shunt and reached the head [16]. The same report mentioned the possibility of severe FES due to the release of large amounts of fat droplets after severe and multiple fractures. Large amounts of fat droplets may have been released early in the onset of the disease in the present case. Also, as a condition specific to our patient, pulmonary hypertension due to interstitial pneumonia may have pressurized the fat droplets, causing them to pass through the pulmonary capillaries prematurely and be dispersed to the head.

**Table 2:** Reported fulminant fat embolism syndrome cases reported in the past developed within 3 h of injury In reported fulminant fat embolism syndrome cases reported in the past, seven cases, including our case, developed within 3 h of injury, and our case developed the earliest after the injury. Most of the cases were severe, and no case of PFO patency was noted by echocardiography or transesophageal echocardiography.

No.	Age/sex	Latent period	Trauma	Presence or absence of PFO	Neurological outcome
1 (Our case)	66/F	49 min	left femoral neck fracture and left sacral bone fracture	None (transthoracic)	GCS: E3V1M4 mRS5
2. Tsuru et al. [13]	83/M	55 min	left distal femoral fracture	None (transesophageal)	Comatose
3. Chen et al. [14]	31/M	1 h	right femoral fracture and distal tibiofibular fracture	None (transthoracic)	Comatose
4. Hirata et al. [15]	85/F	1 h	left femoral neck fracture and left clavicle fracture and fibular fracture	None (autopsy)	Dead at 3 h 20 min after trauma
5. Berlot et al. [16]	17/M	2 h	right open femoral fracture and tibia fracture	None (transthoracic)	Brain death (Day 2)
6. Yang et al. [17]	29/F	2 h 30 min	left femoral fracture and base of the left fifth metatarsal bone fracture	Not listed	Recovered consciousness after 29 days in a coma. Returned to work 6 months after trauma.
7. Eriksson et al. [18]	54/M	2 h 53 min	multiple fractures (rib, clavicle, femoral), pulmonary contusion, and pneumothorax	None (transesophageal)	Brain death (Day 5)

## Conclusions

We described a case of fulminant FES that developed 49 min after a femoral neck fracture and a sacral fracture. The patient developed FES early after the trauma and had a severe course. The patient’s pulmonary hypertension due to interstitial pneumonia may have pressurized the fat droplets, causing them to pass through the pulmonary capillaries prematurely and be dispersed to the head. The possibility of fulminant FES should be considered as a cause of early impaired consciousness after a fracture.
